# Spoligotyping and whole-genome sequencing analysis of lineage 1 strains of *Mycobacterium tuberculosis* in Da Nang, Vietnam

**DOI:** 10.1371/journal.pone.0186800

**Published:** 2017-10-19

**Authors:** Minako Hijikata, Naoto Keicho, Le Van Duc, Shinji Maeda, Nguyen Thi Le Hang, Ikumi Matsushita, Seiya Kato

**Affiliations:** 1 Department of Pathophysiology and Host Defense, The Research Institute of Tuberculosis, Japan Anti-Tuberculosis Association, Kiyose, Tokyo, Japan; 2 The Research Institute of Tuberculosis, Japan Anti-Tuberculosis Association, Kiyose, Tokyo, Japan; 3 Da Nang Lung Hospital, Da Nang, Vietnam; 4 Hokkaido Pharmaceutical University School of Pharmacy, Sapporo, Hokkaido, Japan; 5 NCGM-BMH Medical Collaboration Center, Hanoi, Vietnam; St Petersburg Pasteur Institute, RUSSIAN FEDERATION

## Abstract

**Background:**

Spacer oligonucleotide typing (spoligotyping), a widely used, classical genotyping method for *Mycobacterium tuberculosis* complex (MTBC), is a PCR-based dot-blot hybridization technique to detect the genetic diversity of the direct repeat (DR) region. Of the seven major MTBC lineages in the world, lineage 1 (Indo-Oceanic) mostly corresponds to the East African–Indian (EAI) spoligotype family in East Africa and Southeast Asia.

**Objectives:**

We investigated the genomic features of Vietnamese lineage 1 strains, comparing spoligotype patterns using whole-genome sequencing (WGS) data.

**Methods:**

*M*. *tuberculosis* strains isolated in Da Nang, Vietnam were subjected to conventional spoligotyping, followed by WGS analysis using a high-throughput sequencer. Vietnamese lineage 1 strains were further analyzed with other lineage 1 strains obtained from a public database.

**Results:**

Indicating a major spoligotype in Da Nang, 86 (46.2%) of the 186 isolates belonged to the EAI family or lineage 1. Although typical EAI4-VNM strains are characterized by the deletion of spacers 26 and 27, 65 (75.6%) showed ambiguous signals on spacer 26. *De novo* assembly of the entire DR region and *in silico* spoligotyping analysis suggested the absence of spacer 26, and direct sequencing revealed that the 17^th^ spacer sequence not used for conventional typing, was cross-hybridized to the spacer 26 probe. Vietnamese EAI4-VNM, other EAI-like strains, and those showing a non-EAI pattern lacking many spacers formed a monophyletic group separate from other EAI families in the world.

**Conclusion:**

Information about the alignment of spacers in the entire DR region obtained from WGS data provides a clue for the determination of experimentally ambiguous spoligo patterns. WGS data also helped to analyze the hidden relationships between apparently distinct spoligo patterns.

## Introduction

The methods of genotyping *Mycobacterium tuberculosis* complex (MTBC) facilitate many aspects of tuberculosis studies. The MTBC was previously considered genetically monomorphic in nature but the development of genotyping methods that discriminate strains into distinct lineages has demonstrated previously unrecognized diversity. Consequently, the genetic variations of MTBC have been investigated extensively in phylogenetic studies to understand the evolution and spread of MTBC [1, 2]. Based on the regions of difference (RD) classification system using large sequence polymorphisms (LSP), six major global MTBC lineages have been defined (1 Indo-Oceanic, 2, East-Asian including Beijing, 3 East-African-Indian, 4 Euro-American, 5 West Africa or *Mycobacterium africanum* I, 6 West Africa or *M*. *africanum* II) [3], and another phylogenetic lineage of MTBC has recently been described in Ethiopia as lineage 7 [4]. Whole-genome sequencing (WGS) technology using a high-throughput sequencer has enabled us to obtain more complete information about phylogenetic markers, and SNP-based barcode system is now used to classify a number of sublineages [2].

Among the molecular markers traditionally used, clustered regulatory interspaced short palindromic repeats (CRISPR)-based spacer oligonucleotide typing (spoligotyping) [5] and insertion sequence (IS)*6110*-restriction fragment length polymorphism [6] have often been applied in epidemiologic research. The direct repeat (DR) region of the *M*. *tuberculosis* genome consists of 36 base pair (bp) DR copies and 35 to 41 bp spacers. Each pair of DR and the adjacent spacer is called a direct variant repeat (DVR). The original spoligotyping method uses 43 of 68 DVRs in the region and detects the presence or absence of these spacers by a PCR-based dot-blot hybridization technique [5, 7]. Microbead techniques are also applied to the typing [8, 9]. Alignment of the spacers is well conserved, and the genetic diversity in the region is mostly due to deletion of DVR [7]. International databases for the spoligotypes of these 43 spacers have been developed; SpolDB4 contains data from 40,000 MTBC isolates from 120 countries [10], and the publicly available online database SITVITWEB has over 60,000 isolates from 150 countries [11]. Although spoligotypes are not always consistent with phylogenetic groups [12], some of the spoligotype families are relatively unique. For instance, the East African–Indian (EAI) family strains belong to lineage 1 [13], and the Beijing family strains belong to lineage 2. Thus, conventional spoligotyping system is still informative and practically useful in countries or areas where a variety of MTBC lineages and sublineages coexist.

Spoligotype-defined EAI family strains belonging to lineage 1 are prevalent in East Africa, South Asia, and Southeast Asia [14, 15]. Of these, the EAI4-VNM subfamily is known to be one of the typical Vietnamese *M*. *tuberculosis* genotypes [10, 11], but the relationship between lineage 1 strains and the EAI4 subfamily has not been fully elucidated. In the present study, we explored these genetic features in central Vietnam and compared classical spoligotyping results with WGS.

## Materials and methods

### *M*. *tuberculosis* strains

Cultured *M*. *tuberculosis* isolates were collected from 186 patients with active pulmonary tuberculosis in Da Nang, Vietnam between January 2015 and November 2016. The study was approved by the Ethics Committee for Biomedical Researches, National Hospital of Pediatrics, Vietnam. Written informed consent was collected from all study participants. Genomic DNA was extracted using Isoplant kits (Nippon Gene, Tokyo, Japan). Beijing and non-Beijing strains were discriminated by a single nucleotide variation (SNV) at position 779,615 of H37Rv (AL123456) [16] as described previously [17], and lineage 1 strains were further identified in the non-Beijing group by detecting SNV at position 649,345 [18] using real-time PCR.

### Spoligotyping

Spoligotyping was performed according to a standard protocol [5, 19]. Classification of the spoligotype family was based on the international database SITVIT [11]. Spoligotype patterns characterized by the absence of spacers 26, 27, 29 to 32, and 34 and the presence of 33, but not registered in the database, were regarded as EAI4-like strains in the present study.

### WGS analysis of *M*. *tuberculosis* isolates

A library for WGS analysis was prepared from 200 ng of genomic DNA with the TruSeq Nano DNA LT Sample Preparation Kit (Illumina, San Diego, CA, USA). To improve the amplification of GC-rich sequences of the *M*. *tuberculosis* genome, a PCR step in the library preparation was performed using KOD FX Neo (Toyobo, Osaka, Japan). Paired-end (2 × 250 bp or 2 × 300 bp) sequencing was performed using MiSeq (Illumina).

Paired-end fastq files were used for *de novo* assembly using the Platanus trimming tool-1.0.7 and Platanus_assmbler-1.2.4 [20], and their quality was evaluated using QUAST 4.3 [21]. Contig sequences including the DR region were selected from gap-closed fasta files, and alignment of the spacers was further analyzed using Genetyx-Mac (Genetyx, Tokyo, Japan). Fastq files were also subjected to *in silico* spoligotyping using the SpolPred [22] or SpoTyping-v2.1-commandLine [23] tool. To identify the presence or absence of 68 spacer sequences in fastq files *in silico*, a spacer-EX.fasta file with 68 probes, consisting of 25 nucleotides each, was prepared (**S1 Fasta file**) and served for the standard nucleotide BLAST+ program in the extended SpoTyping tool. The BLAST+ program (ncbi-blast-2.4.0+) was used to further search for nucleotide matches between the 43 original spoligotyping probes and the otherwise unused spacer sequences. The presence or absence of region of difference (RD) 239 was assessed by RD-Analyzer [24] and used to determine lineage 1, and IS*6110* insertion sites were identified by ISMapper [25].

### Sanger sequencing of DNA fragments hybridized with spacer 26 probe by spoligotyping

After hybridization, a membrane with a weakly positive signal at position 26 was excised, briefly washed, immersed in 1× PCR buffer, and heated at 95°C for 10 min. Eluted DNA was re-amplified by PCR with DRa and DRb primers with additional adapter sequences (underlined) at their 5′ end (R1-Dra, 5′-CTGGAGTTCAGACGTGTGGTTTTGGGTCTGACGAC-3′; R2-DRb, 5′-CTCTTTCCCTACACGACCCGAGAGGGGACGGAAAC-3′). The amplified products were subjected to direct sequencing with primers R1 (5′-CTGGAGTTCAGACGTGT-3′) or R2 (5′-CTCTTTCCCTACACGACC-3′) using the BigDye Terminator v3.1 Cycle Sequencing Kit (ThermoFisher Scientific, Waltham, MA, USA) and a 3500xl Genetic Analyzer (ThermoFisher Scientific).

### Sequences of lineage 1 strains downloaded from public databases and phylogenetic analysis

Fastq files of lineage 1 strains reported in Asia and Africa were randomly downloaded from public databases. [26–28]. When their lineage information was unknown, lineage-specific mutations were identified using TB Profiler [29]. As a result, WGS data from 43 lineage 1 strains were obtained and subjected to the *in silico* spoligotyping methods SpolPred and SpoTyping (**S1 Table**). In addition, sequencing reads of Da Nang isolates and the downloaded reads were mapped to the *M*. *tuberculosis* reference genome H37Rv (AL123456), and SNVs were called using CLC Genomics Workbench 9.5 (QIAGEN, Hilden, Germany), with the following parameters: minimum coverage = 10; minimum central quality (Phred score) = 20; and minimum neighborhood quality = 15. After removal of SNVs in the PE/PPE/PGRS genes, concatenated SNVs were obtained and a phylogenetic tree was constructed using RAxML version 8.2.8 [30], with a maximum likelihood search and 100 rapid bootstrap analyses, and then visualized with FigTree v1.4.3 [https://github.com/rambaut/figtree/releases] using *M*. *canettii* (ERR313114) as an outgroup.

## Results

### Characterization of *M*. *tuberculosis* isolates in Da Nang, Vietnam using spoligotyping

Of the 186 Da Nang isolates, 63 (33.9%) Beijing and 123 (66.1%) non-Beijing isolates were identified, and 86 of the 123 isolates belonged to lineage 1. Genomic DNA samples from the 86 Vietnamese lineage 1 isolates were subjected to spoligotyping. Of these, 74 spoligotypes were consistent with or very similar to EAI patterns, which were characterized by the absence of spacers 29 to 32 and 34 and the presence of spacer 33 [10, 31]. The remaining three lacked spacer 33, and nine had larger spacer deletions, showing non-EAI spoligo patterns.

### Ambiguous spoligotypes observed in lineage 1 strains

**Fig 1:**
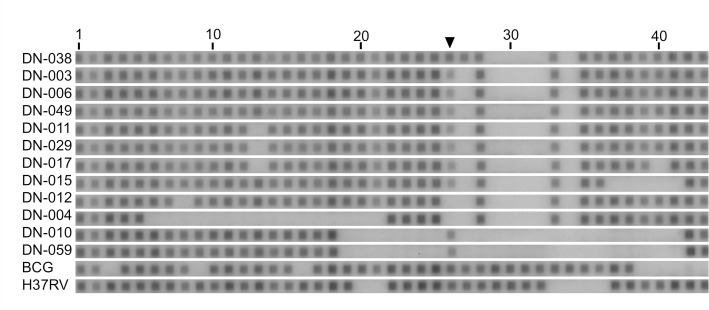
Membrane hybridization patterns using conventional spoligotyping probes. DN-038 to DN-059 were representative lineage 1 isolates from Da Nang. BCG and H37Rv strains were used as controls. The density of dots at position 26 (▼) was weak compared to other positive dots in many isolates.

Among those showing EAI spoligo patterns, typical EAI4-VNM strains are characterized by the additional deletion of spacers 26 and 27 [10, 31]. As shown in **Fig 1**, the hybridization signal of spacer 26 was weak but visible in most of the Da Nang strains. Depending on the assessment of spacer 26, i.e., positive or negative, it was possible to assign two different octal codes and shared international type (SIT) numbers, such as SIT139 in EAI4-VNM and SIT152 (= SIT139 + positive spacer 26) in EAI5 (**Table 1**). To investigate the possible cause of this ambiguity, we selected 12 isolates that served for WGS analysis.

**Table 1:** Ambiguous spoligotyping results from lineage 1 strains in Da Nang.

Weak signals of Spacer 26 (assessed as negative)				Weak signals of Spacer 26 (assessed as positive)		
Octal code	SIT	Clade^a^	No. of isolates	Octal code	SIT	Clade^a^
777777774413771	139	EAI4_VNM	33	777777776413771	152	EAI5
777777000000011	405	ZERO	8	777777002000011	619	unknown
777777777413771	236	EAI5	6	^b^		
777777774413711	456	EAI4_VNM	5	777777776413711	ORPHAN	EAI5
777737774413771	564	EAI4_VNM	4	777737776413771	617	EAI5
777777777413371	234	EAI5	4	^b^		
777737777413771	618	EAI5	3	^b^		
777737777413371	unknown	unknown	3	^b^		
777737774413731	2722	EAI4_VNM	2	777737776413731	2346	EAI1-SOM
777777774403771	ORPHAN	unknown	2	777777776403771	unknown	unknown
775777774413771	unknown	unknown	1	^c^		
760000074413771	unknown	unknown	1	^c^		
777777774413701	unknown	unknown	1	777777776413701	unknown	unknown
763777777413771	792	EAI5	1	^b^		
741737777413771	unknown	unknown	1	^b^		
777777777403771	458	unknown	1	^b^		
777777774413011	unknown	unknown	1	777777776413011	unknown	unknown
777777774410771	unknown	unknown	1	777777776410771	unknown	unknown
577777774413771	1731	EAI4_VNM	1	577777776413771	unknown	unknown
777777774413731	514	EAI4_VNM	1	777777776413731	unknown	unknown
777737774413700	unknown	unknown	1	777737776413700	unknown	unknown
777603000000011	802	ZERO	1	777603002000011	unknown	unknown
777776774413771	unknown	unknown	1	777776776413771	unknown	unknown
777601774413771	622	EAI4_VNM	1	777601776413771	unknown	unknown
777777774412771	unknown	unknown	1	777777776412771	unknown	unknown
777777774413071	unknown	unknown	1	777777776413071	unknown	unknown

SIT, shared international type.

^a^Spoligotyping defined lineages/sublineages according to SITVITWEB [11].

^b^Spacer 26 exhibits a clear positive signal, and the spoligotype is unambiguous.

^c^Spacer 26 exhibits a clear negative signal, and the spoligotype is unambiguous.

### Genomic analysis using a *de novo* assembled sequence of the DR region

**Fig 2:**
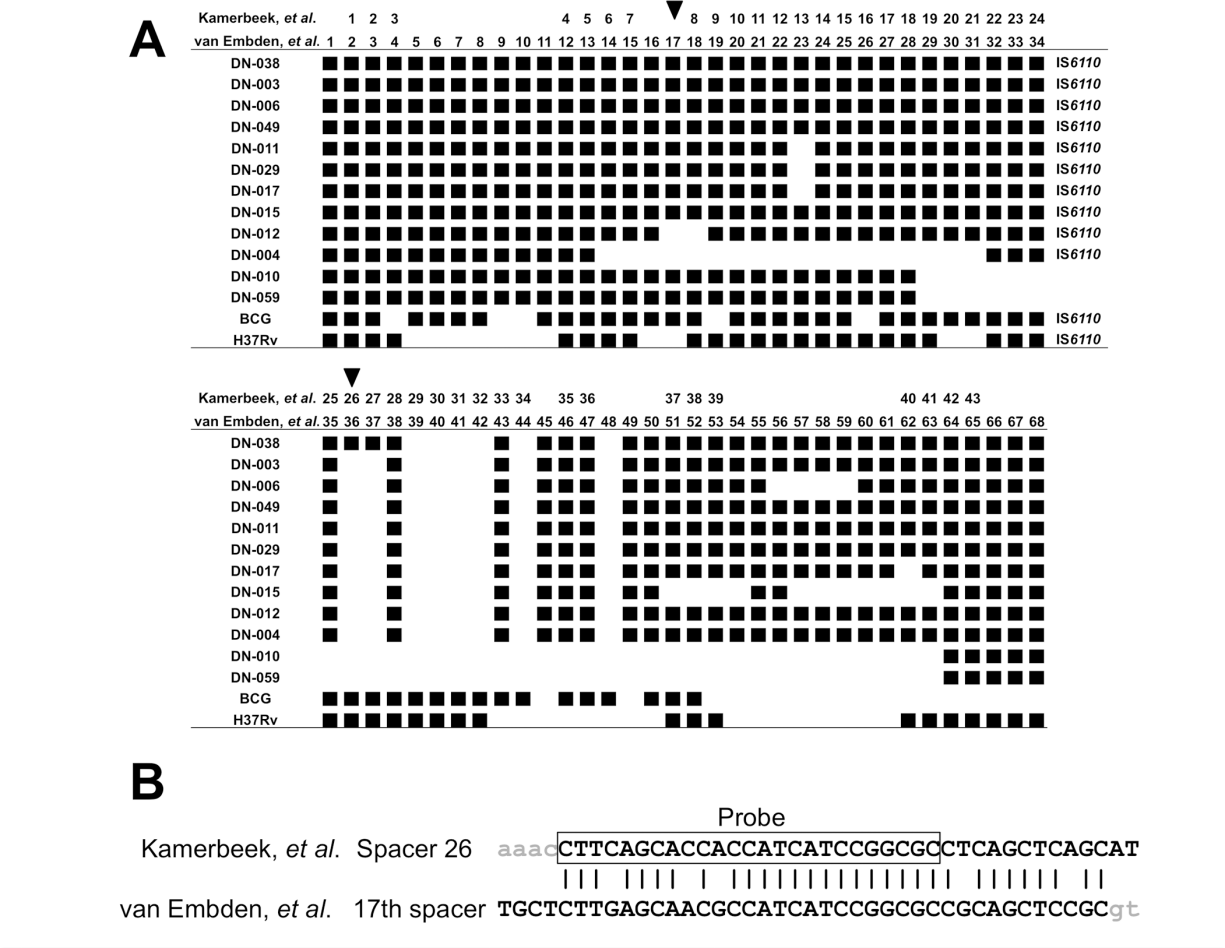
Presence/absence of 68 spacers in *de novo* contig sequences and probe 26 sequence similarity. (A) The presence and absence of 68 spacer sequences are shown. Data from DN-038 to DN-059, 12 isolates from Da Nang, were derived from the *de novo* contig sequences. Data from BCG and H37Rv were obtained from their complete genomic sequences, AM408590.1 and AL123456.2, respectively. The present spacer is shown by a closed square (■). The positions of Kamerbeek’s spacer 26 [5] and van Embden's 17^th^ spacer [7] are marked (▼). (B) The degree of sequence identity between Kamerbeek’s spacer 26 [5] and van Embden's 17^th^ spacer [7]. The oligonucleotide sequence for the spacer 26 probe is boxed in the figure; 22/25 nucleotides (88%) are identical to those of van Embden’s 17^th^ spacer. Nucleotides in the adjacent DR sequences are shown in small grey letters.

*De novo* assemblies resulted in gap-closed fasta files comprising 58–79 contigs of 500 bp or longer (**S2 Table**). Of these, one or two contig sequences containing the entire DR region were identified and examined for the presence or absence of 68 spacers, consisting of 43 used and 25 not used, though previously reported, for conventional spoligotyping [7]. The alignment of these 68 spacers was identical to that shown in a previous report, and the assembled contigs did not hold any novel spacer sequences (**Fig 2A**). The spacer 26 sequence in the original description of spoligotyping [5] was found in the contig of the EAI5 isolate and showed a strong signal at spacer position 26 but was not found in other Vietnamese lineage 1 isolates that had weak signals at position 26. Because these isolates carried 13–24 additional spacer sequences not used for conventional typing, we hypothesized that the ambiguous signal observed at spacer position 26 might be because of cross-hybridization with one of the extra spacer sequences co-amplified by PCR. Based on a sequence similarity search, a nucleotide sequence of the 17^th^ spacer, reported by van Embden *et al*. [7], but not used for the 43-spacer spoligotyping, was 88% (22/25 nucleotides) identical to the spacer 26 typing probe [5] (**Fig 2B**). The strength of similarity of the spacer 26 typing probe to van Embden’s 17^th^ spacer [7], when assessed by e-value and bit-score of BLAST+, was prominent among all comparisons between the 43 original probes and the otherwise unused spacer sequences (**S3 Table**). Two EAI4-like isolates (DN-012 and DN-004) that lacked the 17^th^ spacer in the assembled contig (**Fig 2A**) did not show any ambiguous hybridization signal at position 26 in the conventional typing (**Fig 1**).

### Experimental detection of the DNA sequence cross-hybridized to the spacer 26 probe

In DN-038 and DN-049, DNA fragments hybridized to the membrane of spacer 26 were eluted and re-amplified by PCR. Direct sequencing revealed that the PCR product from DN-049 had the exact nucleotide sequence as the 17^th^ spacer described by van Embden *et al*. [7] **(S1 Fig)**, whereas the product from DN-038 failed to be assessed because of the presence of mixed bases.

### *In silico* spoligotyping and characteristics of Vietnamese lineage 1 isolates

As shown in **S1 Table**, *in silico* spoligotyping of 43 spacers using the short reads of WGS showed no discordance between SpolPred and Spotyping when analytical conditions were optimized. The DN-038 isolate had SNV (position 3,120,278 of H37Rv, AL123456) in the probe region of spacer 28. It was not detected in a setting to identify a complete match. When the Spotyping program was extended to detect the presence or absence of the full set of the 68 spacer sequences reported by van Embden *et al*. [7], the signal patterns were perfectly matched with those obtained by the *de novo* assembly (**S1 Table and Fig 2**). Encouraged by these reproducible results from *in silico* typing, 43 downloaded sequences of lineage 1 strains were also subjected to extended Spotyping, and the patterns of the 68 spacers were compared with those of the Da Nang strains. As a result, the absence of spacers 26 and 27 by the numbering of Kamerbeek *et al*. [5] was unique to Vietnamese EAI4 isolates, and Vietnamese isolates showing the SIT405 pattern (ZERO) in conventional typing revealed a large deletion spanning 35 of the 68 spacer regions (**S1 Table**). As expected, absence of RD239 sequence, a specific marker of lineage 1 strains, was observed in EAI4-VNM, other EAI-like strains, and those showing the SIT405 pattern analyzed in Da Nang (n = 12), as well as in other EAI family strains extracted from the public database (n = 43) (**S1 Table**).

When the IS*6110* sequence was searched in the short reads using ISMapper, at most one IS*6110* was detected in the Vietnamese EAI4 and EAI5 isolates, whereas isolates showing the SIT405 pattern did not have any detectable IS*6110* sequences (**S1 Table**). We confirmed that only one IS*6110* was detected from a complete genome sequence of a Hanoi strain with the EAI-VNM pattern, recently deposited by our group as accession number AP018033 (data not shown) [32].

### *In silico* spoligotyping of lineage 1 strains from other geographical regions

Of 43 lineage 1 strains from the public database, 38 belonged to one of the following clades: EAI1-SOM, EAI2-Manila, EAI2-Nonthaburi, EAI3-IND, EAI4-VNM, EAI5, or EAI6-BGD. The remaining five were non-EAI strains with deletions of many spacers, as determined by WGS analysis using the Spotyping and SpolPred tools (**S1 Table**).

### Phylogenetic analysis

**Fig 3:**
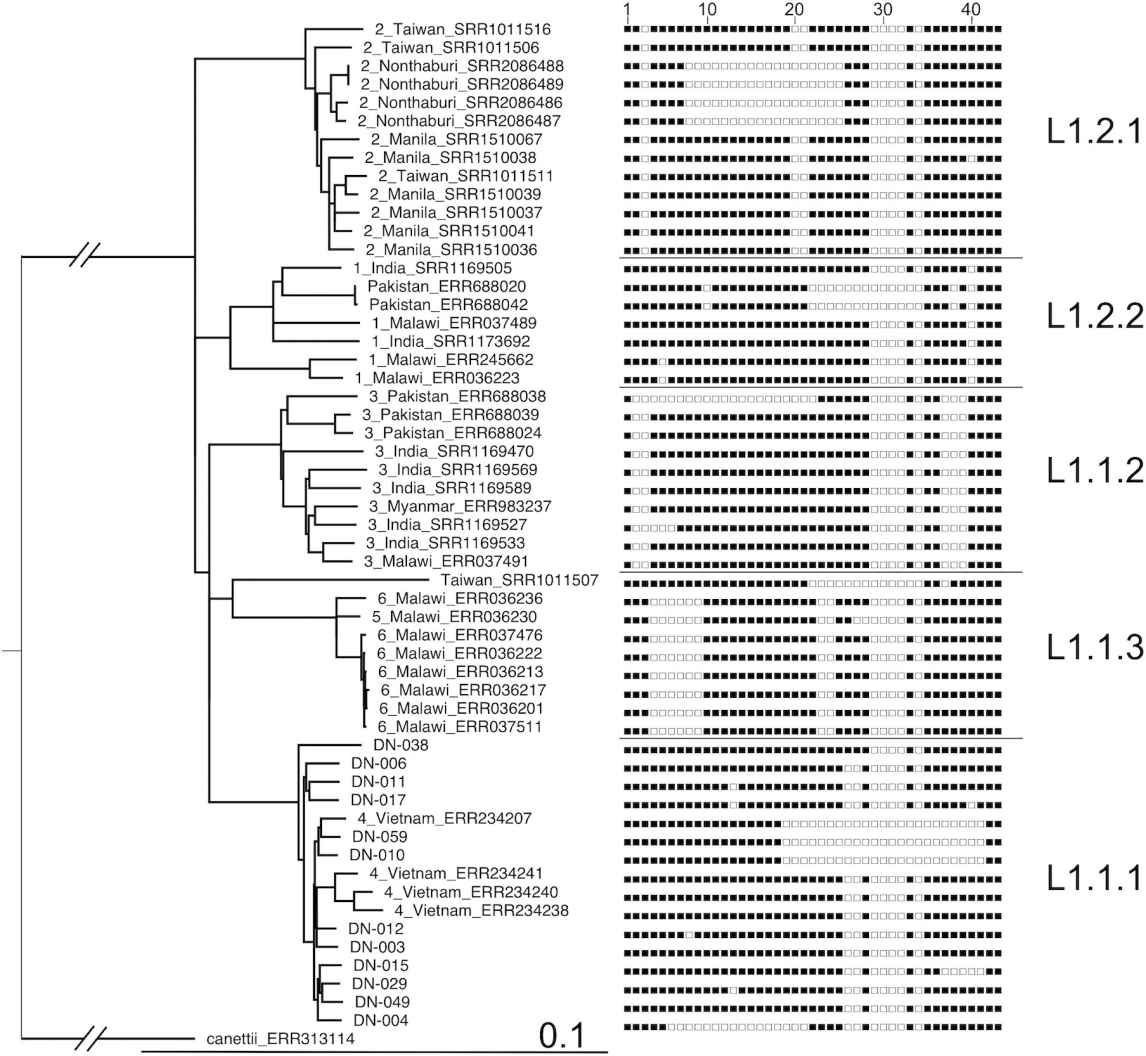
Phylogenetic tree of lineage 1 strains comprising the 12 isolates from Da Nang and 43 from the public database. A phylogenetic tree based on concatenated SNVs from lineage 1 strains (the 12 isolates from Da Nang and 43 from the public database) was constructed using RAxML. *M*. *canettii* was used as an outgroup. The tree was visualized using FigTree v1.4.3. A consensus pattern of spoligotype predicted by *in silico* spoligotyping programs Spotyping [23] and SpolPred [22] is shown on the right side of each isolate. The scale bar indicates the number of substitutions per site. Lineage classification by TB Profiler is also shown.

When a phylogenetic tree was constructed with concatenated SNVs of the Da Nang *M*. *tuberculosis* isolates analyzed in the present study and with other lineage 1 strains from the public database, Vietnamese EAI-VNM formed a closely related group, the L1.1.1 clade including L1.1.1.1 by Coll *et al*. [2], which was discrete from the EAI strains of other geographical regions in Asia and Africa (L1.1.2, L1.1.3, L1.2.1, and L1.2.2). Non-EAI isolates showing the SIT405 pattern and Vietnamese EAI5 were phylogenetically included in the L1.1.1 (EAI4-VNM) group (**Fig 3**), two non-EAI strains (SIT346) from Pakistan were included in the L1.2.2 (EAI1-SOM) group, and one non-EAI strain (SIT1391) from Taiwan was included in L1.1.3 (EAI6-BGD) group.

## Discussion

In the present study, we used spoligotyping and WGS to analyze *M*. *tuberculosis* isolates of lineage 1 from Da Nang, Vietnam. EAI4-VNM and the patterns similar to EAI4-VNM, were major spoligotypes that formed a distinct clade, together with Vietnamese EAI5 and non-EAI (SIT405) spoligotype strains, in a phylogenetic tree. They were separated from lineage 1 strains currently observed in other Asian and African regions.

The EAI family in spoligotyping was originally defined by the absence of spacers 29 to 32 and 34 and the presence of spacer 33 [10, 31] and was found to be distributed among countries in east Africa, south Asia, and Southeast Asia [14, 15]. In a classic spoligotyping analysis, Buu *et al*. reported that EAI family strains were found in 36.3% of 2,207 samples in southern Vietnam [33]. In northern Vietnam, the EAI strains were found in 19.5% of 465 isolates [17]. In southern and northern Vietnam, Beijing strains were also prevalent, whereas in Hue, located at the center of Vietnam, EAI isolates comprised 58.0% of 100 isolates [34]. Da Nang is also located in the center of Vietnam; in the present study, EAI was the most frequent spoligotype (39.8%). Both EAI4-VNM and EAI5 were observed in the above mentioned studies, and we confirmed that EAI strains are still predominant in central Vietnam.

In the present study, we clearly demonstrated that the cross-hybridization between spacer 26 probe and van Embden’s 17^th^ spacer caused ambiguous spoligo patterns, which was initially suspected by the results of WGS analysis using *de novo* assembly and *in silico* spoligotyping tools and further corroborated by DNA elution and amplification using Sanger direct sequencing. The presence of van Embden’s 17^th^ spacer is one of the characteristics of EAI strains [7], and the absence of spacers 26 and 27 is a hallmark of EAI4-VNM. Therefore, cross-hybridization occurs predominantly in the Vietnamese strains. Moreover, the percent sequence similarity between the spacer 26 probe and van Embden’s 17^th^ spacer was prominent among all comparisons between 43 spoligotyping probes and 25 other spacer sequences. Previous Vietnamese studies have shown that EAI isolates have irregular spoligo patterns, namely, the absence of spacers 27, 29 to 32, and 34 and the presence of spacer 26 [34, 35], possibly because of cross-hybridization, as we observed herein. In addition, other studies have also noted that the EAI5 spoligotype SIT152 is closely related to the EAI4 spoligotype SIT139, with a difference of a single positive spacer 26 [36, 37]. Honisch *et al*. found that 10 of 325 *M*. *tuberculosis* strains exhibited discordant spoligotyping results between methods and showed that the presence of spacer 26 in membrane-based results was not reproduced in their MALDI-TOF MS-derived spoligo pattern [38], although the cause of the discrepancy in their study was not revealed.

The EAI family belongs to lineage 1, originally defined by SNV and LSP typing with deletion of RD239 [12, 13]; this finding was confirmed by WGS [2]. According to a recent hypothesis, *M*. *tuberculosis* lineage 1, starting in Africa, has spread in the southern part of Asia as a result of human migration [27]. Presumably reflecting a long evolutionary history, it is conceivable that the EAI family has been divided into several region-specific subgroups apart from their prototype: EAI5 and EAI1-SOM (Somalia); EAI2-Manila (Philippines); EAI2-Nonthaburi (Thailand); EAI3-IND (India); EAI4-VNM (Vietnam); EAI6-BGD/1 and EAI7-BGD/2 (Bangladesh); and EAI8-MDG (Madagascar) [10]. Typical EAI4-VNM is characterized by the additional deletion of spacers 26 and 27 [10, 31]. Although non-EAI strains showing the SIT405 pattern (ZERO) in Da Nang had a large deletion in the DR region with many spacers missing, SIT405 strains were regarded as lineage 1 by LSP in two previous studies [39, 12]. In our WGS analysis, SIT405 strains in Da Nang were phylogenetically very similar to EAI4-VNM, forming a monophyletic group. Non-EAI strains with a series of spacer deletions from Pakistan and Taiwan belonged to the L1.2.2 (EAI1-SOM) and L1.1.3 (EAI6-BGD) clades, respectively. Extensive spacer deletions, including the SIT405 pattern, may have recently emerged across subgroups of the EAI family.

Our study also suggested that the number of IS*6110* sequences was quite different among EAI families. EAI4-VNM strains have only a few IS*6110* sequences, whereas EAI2 strains have more than 10. Roychowdhury *et al*. reported that EAI4 strains have only one IS*6110* sequence [40]; complete genome sequencing of an EAI-VNM strain in Hanoi, Vietnam, has also indicated this [32]. Complete genome sequences derived from a variety of lineages can be used as better references for the mapping of WGS and may contribute to more accurate genotyping in the future.

The spoligotyping method is still useful to discriminate lineage 1 strains from other *M*. *tuberculosis* families, including Beijing strains, particularly in countries where several MTBC lineages are mixed and spread together. Genomic features, including alignment of all spacers in the DR region, should be investigated to determine the correct genotypes. In addition, WGS data may help to analyze the relationships between apparently distinct spoligo patterns. According to recent reports, host responses to *M*. *tuberculosis* are different between ancient MTBC lineages (lineages 1, 5, and 6) and modern MTBC lineages (lineages 2, 3, and 4) [41]. Future progress in genomic research, including lineage 1, will help further the understanding of lineage- or clade-specific phenotypes, diversity of the pathogen, and its interaction with the host.

## Supporting information

S1 Table Summary of isolates included in the analysis. (XLSX)

S2 Table Evaluation of genome assemblies by computing various metrics using QUAST 4.3 with AL123456 as a reference genome. (XLSX)

S3 Table Best nucleotide matches between the 43 original spacer probes and unused spacer sequences, assessed by blastn-short with tabular output format 6. (XLSX)

S1 Fig Electropherogram of bidirectional sequencing of the amplified DNA bound to spacer 26 probe in DN-049 isolate. The 17^th^ spacer sequence reported by van Embden, *et al*. [7] was obtained by direct sequencing using the R2 primer, while the complementary sequence of the 17^th^ spacer was obtained using the R1 primer.
(TIF)

S1 Fasta Fasta file for identification of the presence or absence of 68 spacer sequences in fastq files *in silico* using the extended SpoTyping tool. (FASTA)
