# Supplementary figures and images for: Spoligotyping and whole-genome sequencing analysis of lineage 1 strains of Mycobacterium tuberculosis in Da Nang, Vietnam
Source: PLoS One. 2017 Oct 19;12(10):e0186800. doi: 10.1371/journal.pone.0186800 (PMC5648229; doi:10.1371/journal.pone.0186800)

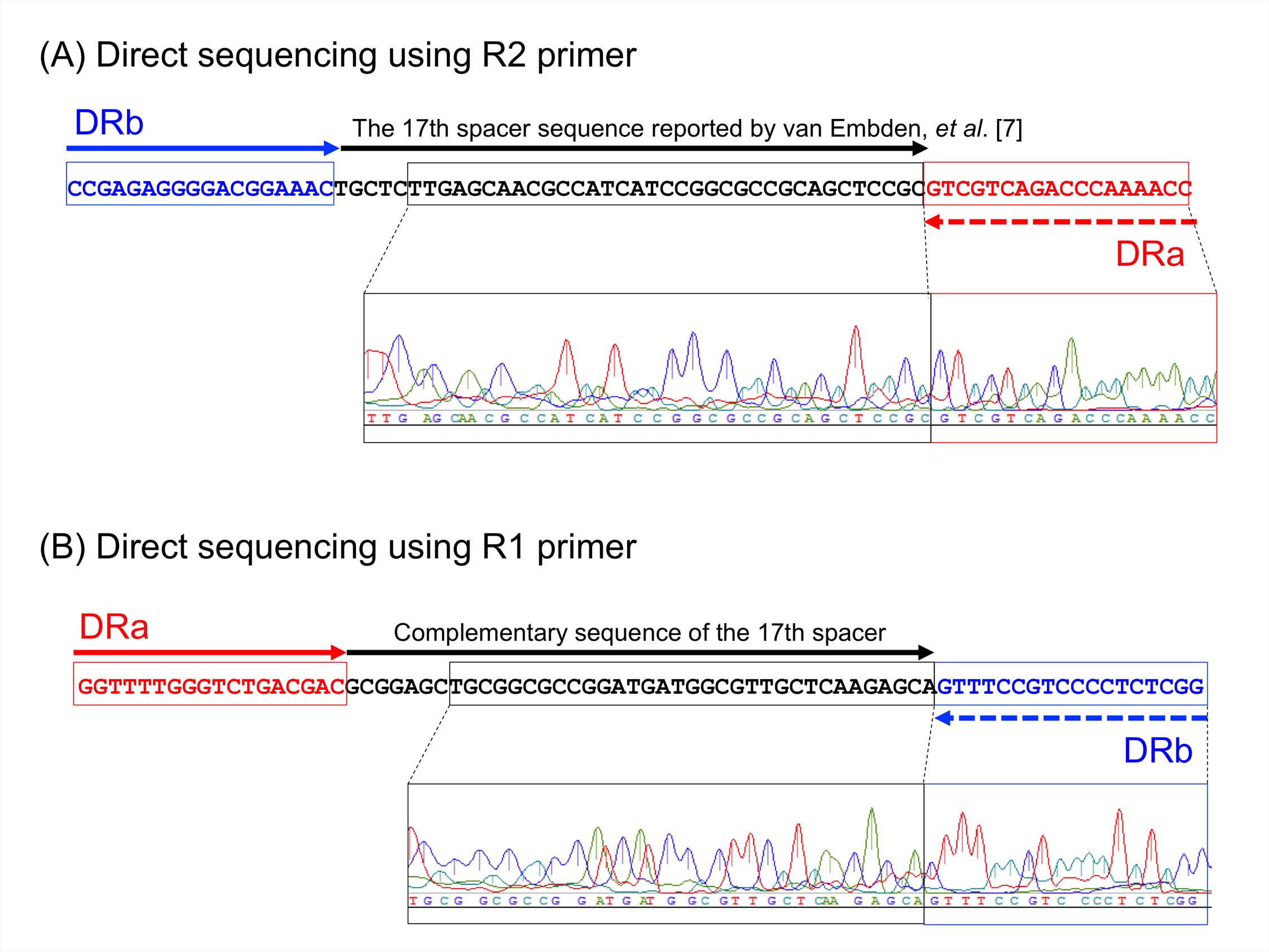

Supplement: S1 Fig — The 17th spacer sequence reported by van Embden, et al. [7] was obtained by direct sequencing using the R2 primer, while the complementary sequence of the 17th spacer was obtained using the R1 primer. (TIF) [file pone.0186800.s004.tif]
